# Dopamine and Risky Decision-Making in Gambling Disorder

**DOI:** 10.1523/ENEURO.0461-19.2020

**Published:** 2020-06-01

**Authors:** Jan Peters, Taylor Vega, Dawn Weinstein, Jennifer Mitchell, Andrew Kayser

**Affiliations:** 1 Department of Psychology, Biological Psychology, University of Cologne, Cologne 50923, Germany; 2 Department of Neurology, VA Northern California Healthcare System, San Francisco, CA 94121; 3 Department of Psychiatry; 4 Department of Neurology, University of California, San Francisco, CA 94143

**Keywords:** dopamine, drift diffusion model, gambling disorder, risky choice

## Abstract

Gambling disorder is a behavioral addiction associated with impairments in value-based decision-making and cognitive control. These functions are thought to be regulated by dopamine within fronto-striatal circuits, but the role of altered dopamine neurotransmission in the etiology of gambling disorder remains controversial. Preliminary evidence suggests that increasing frontal dopamine tone might improve cognitive functioning in gambling disorder. We therefore examined whether increasing frontal dopamine tone via a single dose of the catechol-*O*-methyltransferase (COMT) inhibitor tolcapone would reduce risky choice in human gamblers (*n* = 14) in a randomized double-blind placebo-controlled crossover study. Data were analyzed using hierarchical Bayesian parameter estimation and a combined risky choice drift diffusion model (DDM). Model comparison revealed a nonlinear mapping from value differences to trial-wise drift rates, confirming recent findings. An increase in risk-taking under tolcapone versus placebo was about five times more likely, given the data, than a decrease [Bayes factor (BF) = 0.2]. Examination of drug effects on diffusion model parameters revealed that an increase in the value dependency of the drift rate under tolcapone was about thirteen times more likely than a decrease (BF = 0.073). In contrast, a reduction in the maximum drift rate under tolcapone was about seven times more likely than an increase (BF = 7.51). Results add to previous work on COMT inhibitors in behavioral addictions and to mounting evidence for the applicability of diffusion models in value-based decision-making. Future work should focus on individual genetic, clinical and cognitive factors that might account for heterogeneity in the effects of COMT inhibition.

## Significance Statement

Gambling disorder is associated with impairments in value-based decision-making and cognitive control, functions regulated by the neurotransmitter dopamine. Here, we examined whether increasing frontal dopamine tone via the catechol-*O*-methyltransferase (COMT) inhibitor tolcapone would reduce risky choice in a group of gamblers. Computational modeling did not reveal consistent reductions in risky decision-making under tolcapone in gamblers. If anything, tolcapone increased risky choice. Future work should focus on individual genetic, clinical, and cognitive factors that might account for heterogeneity in the effects of COMT inhibition.

## Introduction

Gambling disorder is a prototypical behavioral addiction that shares behavioral and neural features with substance use disorders (Fauth-Bühler et al., 2017). Consequently, gambling disorder is now classified with substance-related and addictive disorders in the DSM-V (American Psychiatric Association, 2013). Because dysregulation in the dopamine system is implicated in substance use disorders (Robinson and Berridge, 1993; Volkow et al., 2017), similar dysregulation might exist in gambling disorder. Past studies have indeed identified changes in the dopamine system (Clark et al., 2012; Joutsa et al., 2012; Boileau et al., 2013, 2014; van Holst et al., 2018), but there is considerable heterogeneity in the direction of these group differences (Kayser, 2019), and the robustness of some of the reported effects has recently been questioned (Potenza, 2018).

This heterogeneity may partly explain the mixed results of past open-label and placebo-controlled trials of drugs targeting the dopamine system in gambling disorder. While the dopamine D2 antagonist olanzapine was not superior to placebo (Fong et al., 2008; McElroy et al., 2008), both the dopamine D1 receptor antagonist ecopipam (Grant et al., 2014) and the catechol-*O*-methyltransferase (COMT) inhibitor tolcapone (Grant et al., 2013) showed promising results. These different study outcomes could be related to different loci of dopaminergic effects. While olanzapine’s actions are thought to primarily impact striatal function, ecopipam and tolcapone may act more cortically. Tolcapone in particular takes advantage of the fact that significant cortical dopamine inactivation is accomplished via degradation by COMT. Using tolcapone to inhibit COMT could therefore lead to a relatively specific increase in frontal dopamine availability (Käenmäki et al., 2010), thereby augmenting top-down control.

Consistent with this idea, problem gambling is more frequent in gamblers who carry the more active Val/Val polymorphism of the COMT Val158Met allele (rs4680; Grant et al., 2015), presumably leading to lower frontal dopamine tone. Tolcapone also reduced compulsivity in gamblers in proportion to its effect on fronto-parietal activity (Grant et al., 2013) and reduced temporal discounting in gamblers in proportion to its effect on fronto-striatal connectivity (Kayser et al., 2017). Further effects of tolcapone relate to improvements in decision-making and executive control (Farrell et al., 2012; Kayser et al., 2012, 2015; Mitchell et al., 2018).

These domains are generally associated with impairments in gamblers, who show increased temporal discounting (Wiehler and Peters, 2015) and risk-taking (Ligneul et al., 2012; Miedl et al., 2012). In keeping with a dopaminergic influence on these functions, temporal discounting (Pine et al., 2010) and risk-taking (Rutledge et al., 2015; Rigoli et al., 2016) in control subjects are increased following the administration of the dopamine precursor L-DOPA, which is thought to boost dopamine availability more in the striatum than in the cortex (Lloyd and Hornykiewicz, 1972). Overall, however, the human literature is somewhat inconsistent about the direction of these effects (D’Amour-Horvat and Leyton, 2014). We have recently shown that a putative increase in striatal dopamine leads to a reduction in temporal discounting (Wagner et al., 2020), in keeping with rodent work demonstrating that moderate increases in striatal dopamine tend to improve impulse control. Another study only partly replicated the findings of Pine et al. (2010), such that the effects of L-DOPA depended on individual differences in self-control (Petzold et al., 2019). On the other hand, increasing frontal dopamine levels via COMT inhibition might more directly improve decision-making and impulse control, with potential effects of COMT genotype status (Farrell et al., 2012).

Given this hypothesis, we examined a subset of gamblers from a previous randomized, double-blind, placebo-controlled crossover study (Kayser et al., 2017) to assess whether increasing frontal dopamine levels via tolcapone would reduce risk-taking behavior in gamblers. Based on recent work in reinforcement learning (Pedersen et al., 2017; Shahar et al., 2019; Fontanesi et al., 2019; Miletić et al., 2020), temporal discounting (Peters and D'Esposito, 2020; Wagner et al., 2020), and risky choice (Peters and D'Esposito, 2020), we assessed decision-making using a modeling framework based on the drift diffusion model (DDM; Ratcliff et al., 2016) in the context of a hierarchical Bayesian estimation scheme. This modeling approach has the benefit of accounting for the full response time (RT) distributions associated with decisions, thereby providing more detailed information regarding choice dynamics (Pedersen et al., 2017; Miletić et al., 2020) and more stable parameter estimates (Shahar et al., 2019). Furthermore, the DDM can provide novel insights into pharmacological effects on the dopamine system (Wagner et al., 2020). Based on these results, we examined whether a pharmacological modulation of frontal dopamine levels would likewise modulate choice dynamics in frequent gamblers during risky decision-making.

## Materials and Methods

### Participants

Participants were recruited via online advertisements. Subjects with South Oaks Gambling Screen (SOGS) scores >5 (Lesieur and Blume, 1987) were invited to participate in screening procedures. This cutoff has been used clinically to minimize false negatives as opposed to false positives in the diagnosis of gambling disorder (Goodie et al., 2013). To further characterize the extent of their gambling, eligible participants then underwent the Structured Clinical Interview for Pathologic Gambling (Grant et al., 2004), a validated instrument based on DSM-IV criteria.

Subjects were required to be between 18 and 50 years old, in good health, able to read and speak English, and able to provide informed consent. Subjects were excluded if, after completion of the Mini-International Neuropsychiatric Interview (Sheehan et al., 1998), they met screening criteria for an Axis I psychiatric disorder other than gambling disorder, such as major depression, or had a significant medical or psychiatric illness requiring treatment (see also below). Women of reproductive age were required to be using an effective form of contraception and to be neither pregnant nor lactating during study participation. A positive urine drug toxicology screen before any visit was also grounds for exclusion, as was an alcohol level greater than zero as measured by breathalyzer before any visit. Similarly, subjects were excluded for reported use of psychoactive substances (including both prescription medications and drugs of abuse) within the prior two weeks, use of illicit drugs of abuse >10 times in the previous year, or current dependence on marijuana. Subjects could otherwise use marijuana no more than three times per week and were required to refrain from marijuana use for at least 48 h before testing sessions. Subjects who were taking medications with dopaminergic, serotonergic, or noradrenergic actions (although animal work suggests that tolcapone induces increases in dopaminergic but not noradrenergic concentrations; Tunbridge et al., 2004) or who had a known allergy to either tolcapone or the inert constituents in tolcapone capsules, were also excluded. Because tolcapone carries the potential for hepatotoxicity, liver function tests as assessed by phlebotomy were required to be no more than three times the upper limit of normal.

Of the 14 eligible subjects whose data were evaluated here, nine met the criteria for pathologic gambling. Six also met criteria for current alcohol dependence. Because of the strong overlap between gambling disorder and alcohol use disorder, we did not exclude these subjects, but they were required to have a negative breathalyzer test to consent and to participate in all study sessions. All 14 participants had a 0.00 reading on the breathalyzer at the time of consent and at all subsequent study visits. We also did not exclude subjects who used nicotine, and the two regular smokers (out of four total nicotine-using subjects) were both easily able to refrain for the duration of specific study sessions. Table 1 provides an overview of the clinical and demographic data of all participants. The study procedure was approved by the local institutional review board, and participants provided written informed consent before participation.

**Table 1:** Demographic and clinical characteristics of the gamblers

	N/mean (SD)	Range
*N* female/male	6/8	
*N* smokers/nonsmokers	4/10	
COMT genotype (Val/Val, Val/Met, Met/Met)	7/4/3	
Age	32.57 (9.03)	20–47
YoE	14.93 (1.86)	12–18
SOGS	10.79 (3.07)	6–18
GRCS_Total_	97.79 (14.08)	76–116
BDI	11.79 (7.89)	0–27
AUDIT	11.93 (6.40)	2–20
BIS	70.5 (9.62)	50–88

SOGS, South Oaks Gambling Screen (Lesieur and Blume, 1987); GRCS_Total_, Gambling-Related Cognitions Scale (Raylu and Oei, 2004); BDI, Beck Depression Inventory (Beck et al., 1996); AUDIT, Alcohol Use Disorders Identification Test (Saunders et al., 1993); BIS, Barratt Impulsivity Scale (Patton et al., 1995); YoE, years of education; COMT, catechol-*O*-methyltransferase.

### Control group

Following the suggestion by two anonymous reviewers, we compared the data from the gamblers under placebo to data from a set of control participants (*n* = 19) from a previous study (Peters and D'Esposito, 2020). It should be noted, however, that these groups were not matched to the gamblers on age, such that control participants were older on average.

### Drug administration

Subjects were randomized in double-blind, placebo-controlled, crossover fashion to either placebo or a single 200-mg dose of tolcapone on their first visit and the alternative treatment on their second visit. This dose was based on previously published findings that a single 200-mg dose has measurable behavioral effects (Kayser et al., 2012, 2015; Sáez et al., 2015). The present behavioral testing session took place after completion of a functional magnetic resonance imaging (fMRI) study (Kayser et al., 2017). Subjects began the current task ∼3 h after tolcapone and placebo ingestion. Tolcapone is expected to have pharmaco-dynamically relevant serum concentrations for at least 6 h (Dingemanse et al., 1995; Nyholm, 2006) and levels remain markedly above baseline well past 3 h (Jorga et al., 1999, 2000). No subjects reported potential side effects under either the placebo or tolcapone conditions during their participation, and subjects could not reliably differentiate tolcapone from placebo. At the end of each study session, they were asked to guess whether they received tolcapone or placebo. Across the total of 28 choices (14 subjects × two sessions), participants correctly identified tolcapone and placebo 50% of the time (14 choices out of 28).

### Risk-taking task

On each testing day, participants completed 112 trials of a risky-choice task involving a series of choices between a smaller, certain reward ($10 with 100% probability) and larger, but riskier, options. A first set of risky options consisted of all combinations of 16 reward amounts (10.1, 10.2, 10.5, 11, 12, 15, 18, 20, 25, 30, 40, 50, 70, 100, 130, and 150 dollars) and seven probabilities (10%, 17%, 28%, 54%, 84%, 96%, and 99%). We used a second set of probabilities (11%, 18%, 27%, 55%, 83%, 97%, and 98%) in combination with the same series of reward amounts to create a second set of 112 trials. The assignment of the two sets of trials to the two drug conditions was randomized across participants. The experiment was implemented in Presentation (Neurobehavioral Systems). Trials were presented in randomized order and with a randomized assignment of safe/risky options to the left/right side of the screen. Both options remained on the screen until a response was made. An fMRI version of this task has previously been shown to have good test-retest reliability (Peters and Büchel, 2009; Menz et al., 2012) and has been successfully applied to characterize neural correlates of risky decision-making and subjective value in healthy young participants (Peters and Büchel, 2009; Menz et al., 2012).

### Computational modeling

#### Risky choice model

We applied a simple single-parameter discounting model to describe how value changes as a function of probability, such that discounting is hyperbolic over the odds against winning the gamble (Green and Myerson, 2004; Peters and Büchel, 2009; Menz et al., 2012):

(1) $${SV(risky}_{t})=\frac{A_{t}}{1+(\exp\left(h+s*I_{t}\right)\;*\;\theta_{t}},with\;\theta_{t}=\frac{1-p_{t}}{p_{t}}.$$

Here, *A* is the numerical reward amount of the risky option, θ is the odds against winning, and *I* is an indicator variable that takes on a value of 1 for tolcapone data and 0 for placebo data. The model has two free parameters: *h* is the hyperbolic discounting rate from the placebo condition (modeled in log-space), and *s* is a weighting parameter that models the degree of reduction in discounting under tolcapone versus placebo. Thus, the smaller the value of *h*, the smaller the weighting of the odds against winning, and the greater the subjective value of the risky option.

#### Choice rules

We used two different approaches to model participants’ behavior. First, we used softmax action selection to model binary (categorical) decisions. Second, we used the DDM to jointly account for choices and RTs.

#### Softmax action selection

Softmax action selection models the choice probabilities as a sigmoid function of value differences (Sutton and Barto, 1998):

(2) $${P(risky)}_{t}=\frac{e^{\beta *SV({risky}_{t})}}{e^{\beta *SV({risky}_{t})}+e^{\beta *SV({safe}_{t})}}.$$

Here, *SV* is the subjective value of the risky reward according to Equation 1, and β is an inverse temperature parameter, modeling choice stochasticity (for β=0, choices are random and as β increases, choices become more dependent on the option values).

#### Drift diffusion choice rule

To better characterize the dynamics of the decision process, we replaced softmax action selection (Eq. 2) with the DDM, based on recent work in reinforcement learning (Pedersen et al., 2017; Fontanesi et al., 2019; Shahar et al., 2019). The DDM accounts not only for binary choices but for the full reaction time distributions associated with those decisions. We used the Wiener Module (Wabersich and Vandekerckhove, 2014) for the JAGS statistical modeling package (Plummer, 2003) that implements the likelihood function of a Wiener diffusion process. The DDM assumes that decisions arise from a noisy evidence accumulation process that terminates as the accumulated evidence exceeds one of (usually) two decision bounds. Reinforcement learning applications of the DDM have used accuracy coding to define the response boundaries of the DDM (Pedersen et al., 2017; Fontanesi et al., 2019; Shahar et al., 2019), such that the upper boundary corresponds to selections of the objectively superior stimulus, and the lower boundary to choices of the inferior option. This structure is in line with the traditional application of the DDM in the context of perceptual decision-making tasks (Ratcliff and McKoon, 2008). However, in value-based decision-making, there is typically no objectively correct response. Therefore, previous applications of the DDM in this domain have instead re-coded accuracy to correspond to the degree to which decisions are consistent with previously obtained preference judgements (Milosavljevic et al., 2010). This approach is not possible, however, when the goal is to use the DDM to model the preferences that in such a coding scheme would determine the boundary definitions. Therefore, here we applied stimulus coding, such that the upper boundary (1) corresponded to the selection of the risky option and the lower boundary (0) to the selection of the certain option.

We used percentile-based cutoffs for RTs, such that for each participant, the fastest and slowest 2.5% of trials were excluded. Excluding such outlier trials is common practice in the application of the DDM (Pedersen et al., 2017). The reason is that fast outlier trials force the modeled RT distribution to shift as far toward 0 as required to accommodate these observations. This can substantially reduce the goodness-of-fit of the model, because a single outlier RT that is not part of the typical ex-Gaussian-shaped distribution can force the entire distribution to shift, thereby substantially reducing model fit and impacting group-level parameters.

RTs for choices of the certain 100% option were then multiplied by −1 before model estimation. The RT on a given trial is then distributed according to the Wiener First Passage Time (WFPT):

(3) $${RT}_{t}∼WFPT\left(\alpha ,\tau ,z,v\right).$$

Here, α is the boundary separation (modeling response caution and influencing the speed-accuracy trade-off), *z* is the starting point of the diffusion process (modeling a bias toward one of the decision boundaries), τ is the non-decision time (reflecting perceptual and/or response preparation processes unrelated to the evidence accumulation process), and *v* is the drift rate (reflecting the rate of evidence accumulation). In the JAGS implementation of the Wiener model (Wabersich and Vandekerckhove, 2014), the starting point *z* is coded in relative terms and takes on values between 0 and 1. That is, *z *=* *0.5 reflects no bias, *z* > 0.5 reflects a bias toward the upper (risky option) boundary, and *z *<* *0.5 reflects a bias toward the lower (certain option) boundary.

We then compared three variants of the DDM. First, we examined a null model (DDM_0_) without any value modulation. In this model, the four DDM parameters (α,τ, *z*, and *v*) were held constant across trials. Drug effects were modeled by including a term modeling a tolcapone-induced change relative to the placebo condition for each parameter. Second, we examined two previously proposed functions linking trial-by-trial changes in the drift rate *v* to value differences. We examined a linear mapping (DDM_lin_) as previously proposed (Pedersen et al., 2017):

(4) $$v_{t}=v_{coeff}\;*\;\left(SV\left({risky}_{t}\right)-SV\left({safe}_{t}\right)\right).$$

Here, *v_coeff_* maps trial-wise value differences onto the drift rate *v*. *SV* is the subjective value of the rewards according to Equation 1.

We also examined a recently proposed nonlinear (DDM_S_) scheme (Fontanesi et al., 2019):

(5) $$v_{t}=S\left(v_{coeff}\;*\;\left(SV\left({risky}_{t}\right)-SV({safe}_{t})\right)\right).$$

(6) $$S\left(m\right)=\frac{2\;*\;v_{max}}{1+e^{-m}}-v_{max}.$$

Here, *S* is a sigmoid function centered at 0 with *m* being the scaled value difference from Equation 5, and asymptote ± *v_max_*. For DDM_lin_ and DDM_S_, effects of choice difficulty on RTs naturally arise. For more similar values, the trial-wise drift rate approaches 0.

### Hierarchical Bayesian models

Model building proceeded as follows. As a first step, all models were fit at the level of individual participants. We validated that good fits could be obtained, such that posterior distributions were centered at sensible parameter values and the Gelman–Rubin $\hat{R}$ statistic, an estimate of the degree of Markov chain convergence (see below), was in an acceptable range of $1\leq \hat{R}\leq 1.01$. In a second step, models were fit in a hierarchical manner with group-level distributions for all parameters. We used the same convergence criteria as for the single-subject models ($1\leq \hat{R}\leq 1.01$). For group level hyperparameters, we used weakly informative priors (i.e., uniform distributions defined over sensible ranges for means, Gamma distributions for precision). Here, models were fit separately to the data from the placebo and tolcapone conditions, to examine whether drug administration altered the relative model ranking. Finally, after identifying the variant of the DDM that accounted for both the placebo and tolcapone data best, we fit this model across drug conditions. In this final combined model, parameters from the placebo condition were modeled as the “baseline,” and all drug effects were modeled as Gaussians with group level priors with μ=0,σ=2.

### Data availability

Data cannot be shared publicly because participants did not consent to have their data posted in a public repository. Data are available from https://zenodo.org/record/3760335 for researchers who meet the criteria for access to confidential data.

### Code accessibility

JAGS model code is available on the Open Science Framework (https://osf.io/wtg89/). The JAGS model code referenced here is the Extended Data 1.

10.1523/ENEURO.0461-19.2020.ed1 Extended Data 1 JAGS model code of all hierarchical models (DDM0, DDM_lin_ and DDM_S_) is available as extended data at https://osf.io/wtg89/. Model parameters and the input data structure expected by the model are explained in detail in the header of each code file. Download Extended Data 1, ZIP file.

### Model estimation and comparison

Models were fit using Markov Chain Monte Carlo (MCMC) as implemented in JAGS (version 4.2; Plummer, 2003) with the *matjags* interface (https://github.com/msteyvers/matjags) for MATLAB (MathWorks) and the JAGS Wiener module (Wabersich and Vandekerckhove, 2014). For each model, we ran two chains with a burn-in period of 100,000 samples and thinning of 2. A total of 10,000 additional samples was then retained for further analysis. Chain convergence was assessed via the $\hat{R}$ statistic, where we considered $1\leq \hat{R}\leq 1.01$ as acceptable values for all group-level and individual-level parameters. Relative model comparison was performed via the Deviance Information Criterion (DIC), where lower values indicate a better fit (Spiegelhalter et al., 2002).

### Posterior predictive checks

We additionally performed posterior predictive checks to ensure that the best-fitting model captured key aspects of the data. Therefore, during model estimation, we simulated 10,000 full datasets from the hierarchical models based on the posterior distribution of parameters. For each participant and drug condition, model-predicted RT distributions for a random sample of 1000 of these simulated datasets were then smoothed with non-parametric density estimation (*ksdensity.m* in MATLAB) and overlaid on the observed RT distributions for each subject and drug condition.

### Analysis of drug effects

We characterize drug effects in the following ways. First, we show group posterior distributions for all parameters, and 85% and 95% highest density intervals for the posterior distributions of the tolcapone-induced changes in parameters (shift parameters). Additionally, we report Bayes factors (BF) for directional effects (Marsman and Wagenmakers, 2017; Pedersen et al., 2017) based on the posterior distributions of these shift parameters. This value was determined via non-parametric kernel density estimation in MATLAB (*ksdensity.m*) and computed as BF=i/(1-i), where *i* is the integral of the posterior distribution from 0 to +∞. Following common criteria, BF > 3 indicate support for a model, whereas BF > 12 indicate substantial support. Conversely, BF < 0.33 are interpreted as evidence in favor of the alternative model. Lastly, we report standardized effect sizes for all drug-induced changes and group differences, which we calculated based on the means of the group-level posterior mean and precision parameters of the hierarchical model.

### Genetics

DNA extraction and SNP analysis were performed on salivary samples (Salimetrics) collected during the screening visit. DNA was extracted using Gentra Puregene reagents and protocols and quantified using the Pico Green method (Invitrogen/Invitrogen). Genotyping of the COMT (rs4680) polymorphism via polymerase chain reaction was conducted using TaqMan technology (Applied Biosystems).

## Results

### Model-free analyses

**Figure 1.**
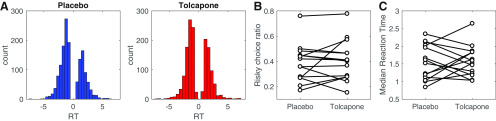
***A***, Overall RT distributions for placebo (blue) and tolcapone (red). Here, positive RTs reflect choices of the risky option, and negative RTs reflect choices of the safe option. ***B***, Proportion of choices of the risky option RT per participant and drug condition. ***C***, Median RT per participant and drug condition.

RT distributions across participants per drug condition are shown in Figure 1*A*. Arcsine square root transformed risky choice ratios (Fig. 1*B*) did not differ significantly between drug conditions (*t*_(13)_ = –0.677, *p* = 0.51, 95% confidence interval (CI): [–0.18, 0.095]). Likewise, median RTs did not differ significantly between drug conditions (*t*_(13)_ = –0.184, *p* = 0.857, 95% CI: [–0.32, 0.27]), arguing that tolcapone did not induce low-level motor effects.

**Figure 2.**
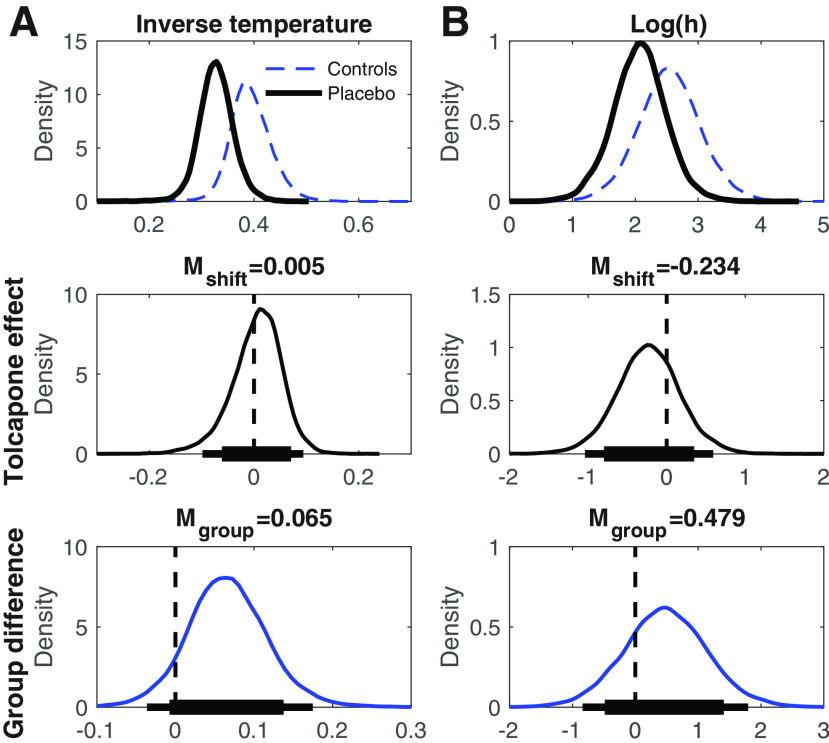
Top row, Group-level posterior distributions for parameter means under placebo (solid black line, ***A***: softmax inverse temperature, ***B***: log(h) [risk-taking]). The dashed blue lines plot the group posterior distributions from the control group of a previous study (*n* = 19; Peters and D'Esposito, 2020). Center row, Group level posterior distributions for tolcapone-induced changes for each parameter. Bottom row, Posterior distributions of group differences between gamblers under placebo from the present study and the control group from Peters and D'Esposito (2020). The thin (thick) horizontal lines in the center and bottom row indicate 95% (85%) highest density intervals.

### Softmax choice rule

In a first step we fit with a hyperbolic probability discounting model (Eq. 1) in combination with softmax action selection (Eq. 2). Posterior distributions under placebo as well as group and tolcapone effects are summarized in Figure 1 and Table 2. Compared with the control group from Peters and D'Esposito (2020), gamblers under placebo if anything showed greater risk taking (BF = 3.59) and greater decision noise (smaller inverse temperature, BF = 9.36). Tolcapone had no detectable effect on decision noise (BF = 1.28) and, if anything, reduced probability discounting in gamblers (BF = 0.384).

**Table 3:** Model comparison of the DDMs, separately for the two drug conditions

Model	Placebo DIC Rank		Tolcapone DIC Rank	
DDM_0_	42,383	3	43,177	3
DDM_lin_	36,136	2	38,302	2
DDM_S_	30,354	1	32,240	1

Under both placebo and tolcapone, the data were best accounted for by a model including a non-linear mapping from trial-wise value differences to drift rates (DDM_S_).

**Table 2:** Summaries of group differences in softmax model parameters and of tolcapone effects on softmax model parameters

Softmax model parameter	Group difference			Tolcapone effect		
	M_diff_	*d*	BF	M_diff_	*d*	BF
Inverse temperature (β)	0.065	0.698	9.36	0.005	0.048	1.28
Log(h)	0.479	0.263	3.59	–0.234	–0.169	0.384

In the summary of group differences in softmax model parameters, for each parameter, we report the mean group difference (controls – gamblers_placebo_), standardized effect sizes (Cohen’s *d*; see Materials and Methods), and BF testing for directional effects (Marsman and Wagenmakers, 2017; Pedersen et al., 2017). BF < 0.33 indicates evidence for a increase in gamblers_placebo_ versus controls, whereas BF > 3 indicates evidence for a reduction (see Materials and Methods). In the summary of tolcapone effects on softmax model parameters, for each parameter, we report the mean change under tolcapone versus placebo, standardized effect sizes (Cohen’s *d*), and BF testing for directional effects. Here, BF > 3 indicates evidence for an increase under tolcapone, whereas BF < 0.33 indicates evidence for a decrease.

**Table 4:** Summaries of group differences in DDM model parameters and of tolcapone effects on DDM model parameters

DDM model parameter	Group difference			Tolcapone effect		
	M_diff_	*d*	BF	M_diff_	*d*	BF
Boundary separation (α)	0.966	1.15	328.0	0.063	0.089	1.81
Non decision time (τ)	0.328	0.784	14.87	–0.003	–0.031	0.815
Starting point / bias (*z*)	–0.011	–0.211	0.403	0.004	0.088	1.47
Drift rate *v* (max)	–0.236	–0.741	0.065	–0.166	–1.84	0.073
Drift rate *v* (coeff)	–0.047	–0.865	0.181	0.069	0.910	7.51
Log(h)	0.575	0.344	4.40	–0.286	–0.281	0.20

In the summary of group differences in DDM model parameters, for each parameter, we report the mean group difference (controls – gamblers_placebo_), standardized effect sizes (Cohen’s *d*; see Materials and Methods), and BF testing for directional effects (Marsman and Wagenmakers, 2017; Pedersen et al., 2017). BF < 0.33 indicate evidence for an increase in gamblers_placebo_ versus controls, whereas BF > 3 indicates evidence for a reduction (see Materials and Methods). In the summary of tolcapone effects on DDM model parameters, for each parameter, we report the mean change under tolcapone versus placebo, standardized effect sizes (Cohen’s *d*), and BF testing for directional effects. Here, BF > 3 indicates evidence for an increase under tolcapone, whereas BF < 0.33 indicates evidence for a decrease.

### Model comparison

We next focused on DDM choice rules, and compared three variants of the DDM: a null model without any value modulation (DDM_0_), a model with a linear scaling of trial-wise drift rates (DDM_lin_) and a model with nonlinear (sigmoid) drift rate scaling (DDM_S_). To ensure that drug condition did not impact model ranking, we first fit the three models separately to the data from the placebo and tolcapone conditions. As can be seen from Table 3, model ranking was the same in the two drug conditions, such that models including value modulation of the drift rate outperformed the DDM_0_, and the nonlinear DDM_S_ fit the data better than the DDM_lin_.

### Initial model validation

**Figure 3.**
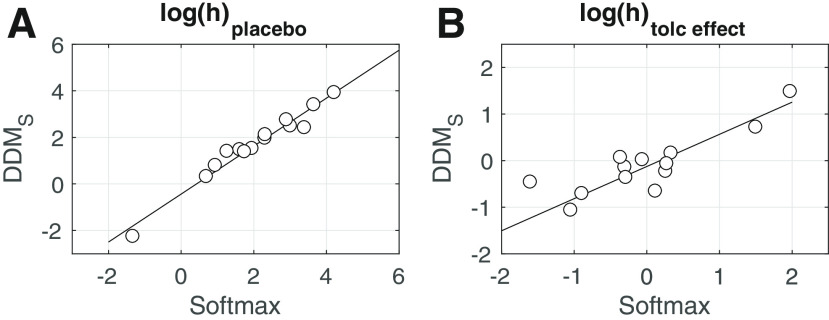
***A***, Correlation between the probability discount rate log(h) under placebo, estimated via standard softmax and via the DDM_S_. ***B***, Correlation between the change in log(h) under tolcapone, estimated via standard softmax and via the DDM_S_.

We next fit the DDM_S_ to the combined data from the two drug conditions, modeling the placebo condition as the baseline, and tolcapone-induced changes in each parameter as additive changes relative to that baseline using Gaussian priors centered at zero. As an initial validation analysis, we checked whether the choice model parameters estimated via a standard softmax choice rule (Eq. 2) could be reproduced using the DDM. We therefore correlated single subject mean posteriors for log(h) (risk taking under placebo) and log(h)*_tolceffect_* (the change in risk taking under tolcapone) from the hierarchical DDM_S_ and the hierarchical model with softmax action selection (see Fig. 3). Both parameters were highly correlated between estimation schemes (log(h): *r *=* *0.98, *p *<* *0.0001, log(h)*_tolceffect_*: *r *=* *0.93, *p *<* *0.0001), indicating that parameters estimated via standard methods could be reproduced using the DDM (Peters and D'Esposito, 2020).

### Posterior predictive checks

**Figure 4.**
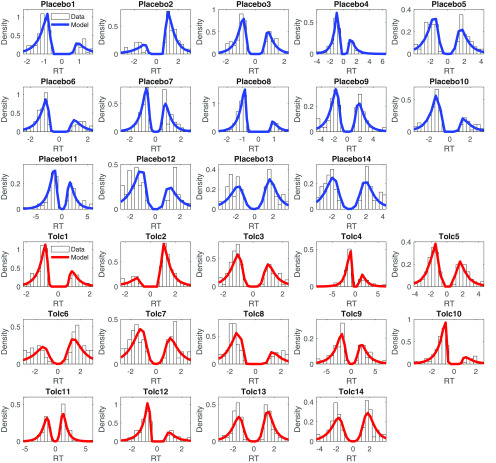
Posterior predictive plots of the drift diffusion temporal discounting model with nonlinear value scaling of the drift rate (DDM_S_) for all 14 participants (blue: placebo, red: tolcapone). Histograms depict the observed RT distributions for each participant. The solid lines are smoothed histograms of the model predicted RT distributions from 1000 individual subject datasets simulated from the posterior of the best fitting hierarchical model. RTs for smaller-sooner choices are plotted as negative, whereas RTs for larger-later choices are plotted as positive. The *x*-axes are adjusted to cover the range of observed RTs for each participant.

Then we examined the extent to which the DDM_S_ could reproduce the reaction time distributions observed in individual participants. To this end, we simulated 10,000 full datasets from the models’ posterior distribution. The histograms in Figure 4 show the observed reaction time distribution for each participant and drug condition, with a smoothed density estimate of the model-generated reaction time distribution (based on 1000 random samples from the simulations) overlaid. Generally, the model accounted reasonably well for the observed reaction time distributions in most participants. The DDM_S_ also accounted for a similar proportion of binary decision under tolcapone and placebo [M[range]_placebo_: 0.899 (0.798–0.962), M[range]_tolcapone_: 0.879 (0.717–0.972), *t*_(13)_ = 1.21, *p* = 0.249].

### Effects of tolcapone on risk-taking and diffusion model parameters

**Figure 5.**
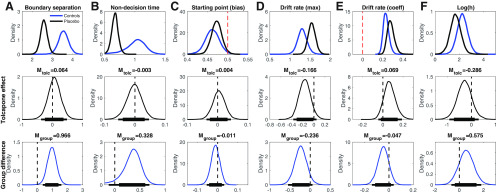
Top row, Group-level posterior distributions for parameter means in the gamblers under placebo (*n* = 14, solid black line) and the Peters and D'Esposito (2020) controls (*n* = 19, solid blue line). ***A***, Boundary separation. ***B***, Non-decision time. ***C***, Bias. ***D***, *v_max_*. ***E***, *v_coeff_*. ***F***, log(h) [risk-taking]. The dashed red line in ***C*** denotes 0.5, i.e., a neutral bias. The dashed red line in ***E*** denotes zero, i.e., no value modulation of the drift rate. Center row, Group level posterior distributions for tolcapone-induced changes for each parameter. Bottom row, Posterior distributions of group differences between gamblers under placebo from the present study and the control group from Peters and D'Esposito (2020). The thin (thick) horizontal lines in the center and bottom row indicate 95% (85%) highest density intervals.

We next examined the posterior distributions of parameters of the final DDM_S_ model in more detail. Figure 5, top row, shows the group level posterior distributions for parameters at baseline (placebo) as well as parameters for the Peters and D'Esposito (2020) control group. Figure 5, center row, shows posterior distributions for tolcapone effects, and the bottom row shows posterior group differences (gamblers_placebo_ vs controls). Mean group differences, tolcapone effects and BF testing for directional effects are listed in Table 4. Under placebo, both boundary separation (response caution; Fig. 5*A*; Table 4) and non-decision time (Fig. 5*B*; Table 4) in the gamblers under placebo were substantially lower than the corresponding values in the control group. Both groups also exhibited a bias toward the safe option, reflected in a posterior distribution of the starting point that was shifted slightly toward zero (Fig. 5*C*). The maximum drift rate *v_max_* at placebo was higher in gamblers versus controls (Fig. 5*D*; Table 4), and there was a robust positive effect of value differences on the trial-wise drift rates, as reflected in a positive drift rate coefficient parameter under placebo (*v_coeff_*; Fig. 5*E*). Interestingly, log(h) (i.e., risk-taking) in the gamblers under placebo (Fig. 5*F*) was higher compared with our previous control group, such that increased risk-taking in gamblers was ∼4.4 times more likely than a reduction. Notably, a log(h) value of 0 would indicate risk neutrality such that the subjective value of a risky option corresponds to its expected value. Both groups were therefore risk averse, but gamblers less so than controls.

All drug effects are summarized in the right columns of Table 4 (mean parameter changes between tolcapone and placebo, standardized effect sizes (Cohen’s *d*), BF for directional effects; see Materials and Methods). The posterior distributions for the tolcapone-induced change for boundary separation (Fig. 5*A*), non-decision time (Fig. 5*B*), and starting point (Fig. 5*C*) were all centered at zero with effect sizes of |*d*| < 0.1. In contrast, under tolcapone, there was evidence for a decrease in the maximum drift rate (*v_max_*; *d* = −1.84, BF = 0.073), an increase in the value-dependent drift-rate modulation (*d *=* *0.901, BF = 7.51) and for a relative increase in risky decision-making as indexed by the hyperbolic discount rate *h* (*d* = –0.281, BF = 0.20). Tolcapone, thus, if anything, shifted risk preferences in the gamblers toward risk neutrality.

### Compensation between drift rate components

Because previous reports suggested a negative association between *v_max_* and *v_coeff_* (Fontanesi et al., 2019), we examined whether there might also be some compensation between these parameters in our data. We therefore ran additional models where we fixed either drift rate component under tolcapone to that parameter’s value under placebo (that is, keeping either parameter constant while allowing the other to vary according to the drug condition). When *v_max_* was fixed to the placebo value, there was no longer any evidence for a drug-induced change in *v_coeff_* (BF = 1.36, as compared with BF = 7.51 in the full model). In contrast, when *v_coeff_* was fixed to the placebo value, the reduction in *v_max_* was still observed, although somewhat attenuated (BF = 0.17 as compared with BF = 0.073 in the full model). Full results from these models are available at OSF (https://osf.io/wtg89/).

### Consistency of tolcapone effects across participants

**Figure 6.**
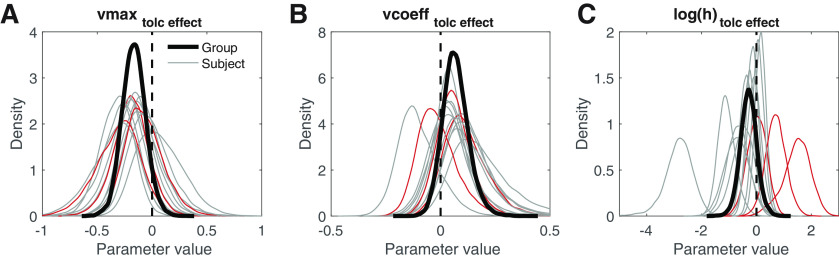
Posterior group means (solid black lines) and individual subject posterior distributions (gray: Val/Val and Val/Met; red: Met/Met) for the tolcapone-induced changes in maximum drift rate (***A***), in value-dependent drift rate modulation (***B***), and in the probability discount rate (***C***). The mean change in *v_max_* was <0 in 13/14 subjects. In *v_coeff_*, it was >0 in 12/14 subjects, and in log(h), it was <0 in 9/14 subjects.

We finally examined the consistency of the latter three group effects across participants by overlaying individual posterior distributions for the tolcapone effects over the average group effects for parameters showing drug effects at the group level (Fig. 6*A*, *v_max_*, *B*, *v_coeff_*, *C*, log(h)). Under tolcapone, 13/14 participants showed a mean reduction in the maximum drift rate *v_max_*, 12/14 showed an increase in the drift rate scaling *v_coeff_*, and 9/14 showed a decrease in log(h) (increase in risk-taking). For transparency, we have highlighted the three Met/Met genotype participants in these plots (red lines), although the analysis of genotype effects is underpowered.

## Discussion

Gambling disorder is associated with impairments in value-based decision-making, including increased temporal discounting and reduced risk aversion (Wiehler and Peters, 2015). Here, we tested whether risky decision-making in gamblers could be attenuated by the COMT inhibitor tolcapone, which predominantly increases dopamine levels in the frontal cortex. Choice data were modeled in a hierarchical Bayesian scheme with the DDM as the choice rule to account for both choices and reaction time distributions. In contrast to our initial hypothesis, if anything tolcapone increased risky decision-making (small effect size) by shifting preferences in gamblers more toward risk neutrality. Examination of the DDM parameters showed a reduction in the maximum drift rate under tolcapone (large effect size) and an increase in the value dependency of the drift rate (large effect size). Together, these results suggest that tolcapone might tie decision-making more tightly to subjective value differences, but that the subjective value of risky options is possibly increased.

We used a modeling scheme based on the DDM, which has recently gained some popularity in reinforcement learning and value-based decision-making (Pedersen et al., 2017; Fontanesi et al., 2019; Shahar et al., 2019; Peters and D'Esposito, 2020; Wagner et al., 2020). As was reported in previous work (Peters and D'Esposito, 2020), choice model parameters estimated via a standard softmax function could be reliably reproduced using the DDM as the choice rule. Posterior predictive checks revealed that the best-fitting DDM reproduced individual subject reaction time distributions reasonably well in both drug conditions. In keeping with previous work on DDM choice rules (Fontanesi et al., 2019; Peters and D'Esposito, 2020), we conducted a model comparison and evaluated both a linear and nonlinear mapping from value differences to trial-wise drift rates. The nonlinear DDM_S_ fit the data better in both drug conditions, confirming previous results of nonlinear drift rate scaling.

The control group was not matched to the gamblers on demographic variables, such that some caution is warranted when interpreting the group differences. However, it is interesting to see that gamblers under placebo exhibited substantially more premature responding than controls (lower boundary separation) as well as faster non-decision times, which could be expected given that increased motor impulsivity is often observed in gambling disorder (Chowdhury et al., 2017). Furthermore, an increase in risky decision-making in gamblers versus controls was ∼4.4 times more likely, given the data, than a reduction, which is in line with previous findings of increased risk-taking in gamblers (Ligneul et al., 2012; Miedl et al., 2012). Notably, both groups were overall risk averse (log(h) was substantially >0), such that gamblers preferences were shifted more toward risk neutrality than controls.

Our results suggest small effects (|*d*| < 0.1) of tolcapone on three parameters of the DDM: boundary separation, non-decision time, and starting point (bias). This finding suggests that overall response caution (as reflected in the boundary separation parameter) and processes related to motor preparation and/or stimulus processing (as reflected in the non-decision time) were largely unaffected by tolcapone. In contrast, there was some evidence that tolcapone modulated drift rate components, and if anything, reduced probability discounting in gamblers, compared with placebo. The latter effect was similarly observed for the standard softmax choice rule and for the DDM. What mechanism might drive the observed effects of tolcapone on risky decision-making and value evidence accumulation? Our approach was motivated by the idea that tolcapone might attenuate risky choice via an augmentation of prefrontal cortex (top-down control) functions. The lateral prefrontal cortex is implicated in cognitive control (Miller and Cohen, 2001; Szczepanski and Knight, 2014), and disruption of prefrontal cortex function can increase risk-taking and impulsivity (Knoch et al., 2006; Figner et al., 2010; Sellitto et al., 2010; Peters and D'Esposito, 2016, 2020). Likewise, tolcapone has been shown to act through an enhancement of prefrontal cortex activation and/or fronto-striatal interactions (Kayser et al., 2012, 2017; Grant et al., 2013). However, although the drug effect on risky choice was small, it was in the opposite direction, increasing risky choice rather than attenuating it. Furthermore, the directionality and effect size of the drug effect on log(h) showed some heterogeneity across participants (Fig. 6*C*). In the absence of task-related imaging data, drawing definite conclusions regarding the mechanism underlying these differential effects of tolcapone on risky choice remains speculative, and individual genetic differences likely contribute to these variable results.

Similarly, it remains unclear through what exact mechanism an increase of frontal dopamine levels might affect the changes in value dependency of the drift-rate observed in the present study. Ventromedial prefrontal cortex is involved in coding for reward valuation during learning and decision-making (Bartra et al., 2013; Clithero and Rangel, 2014). It could thus be speculated that tolcapone might enhance such value representations, thereby increasing the value dependency of trial-wise drift rates. However, at the same time maximum drift rates were reduced under tolcapone, an effect that was consistent across participants (see Fig. 6). Additional analyses revealed that this might in part reflect at a trade-off between *v_max_* and *v_coeff_* parameters in the model, such that reduced *v_max_* can be compensated for by increases in *v_coeff_* under some conditions. Such interactions require further study in the use of diffusion model choice rules in larger samples.

Finally, dopamine has different functions in different prefrontal cortex subregions (Robbins and Arnsten, 2009), such that different dopamine-dependent cognitive functions might exhibit different dose-response functions (Floresco, 2013) and thus be differentially modulated by tolcapone. A thorough assessment of these complexities, including process-dependent baseline effects and potential subregion-specific effects of tolcapone will need to be more fully addressed in future studies (Kayser, 2019).

While we genotyped participants for the COMT Val158Met polymorphism, drawing any conclusions regarding genotype effects in a small sample study such as the present one is obviously highly problematic. On the other hand, not reporting genotype data that is available would also seem inappropriate given the previously suggested COMT genotype dependency of tolcapone effects on risk-taking (Farrell et al., 2012). In their between-subjects study, Farrell et al. (2012) reported increased risk aversion in Val/Val participants under tolcapone, compared with a group of Met/Met carriers. In contrast to that study, in our data set the two participants showing the largest reduction in risky choice under tolcapone were Met/Met carriers. This result is in line with the frequent observation that dopamine effects on cognitive functions mediated by the prefrontal cortex depend on baseline dopamine availability in an inverted U-shaped fashion (Cools and D'Esposito, 2011). However, in this model, Met/Met carriers exhibit a higher frontal dopamine level at baseline due to the COMT enzyme being less active. Further COMT suppression (e.g., via tolcapone) is then thought to move Met/Met subjects into an “overdosed” state, impairing performance relative to placebo (Tunbridge et al., 2006; Cools and D'Esposito, 2011; Farrell et al., 2012). This is not compatible with the substantial reduction in probability discounting observed for 2/3 Met/Met carriers. However, as mentioned above, different cognitive functions might show different functional forms of dopamine baseline dependency (Floresco, 2013), which would require much larger subject numbers to fully evaluate.

There are several additional limitations of the present study that need to be acknowledged. First, given the small sample size, our findings require replication in larger samples and disorders other than gambling disorder. Second, although gender was relatively balanced in the present study, which is often not the case in gambling disorder, we were underpowered to examine sex differences. Third, we did not test a control group specifically matched to the gamblers and rather focused on potential drug effects in this clinical sample. The aim of the project was to examine the degree to which behavioral markers of gambling disorder such as risk-taking and temporal discounting (Kayser et al., 2017) could be improved by COMT inhibition, but future studies could benefit from a more detailed exploration of the effects of COMT inhibition on risk-taking in healthy controls, as done in a previous study for inter-temporal choice (Kayser et al., 2012). However, to provide some reference for risk preferences in our particular sample of gamblers, we have compared their parameters under placebo to a group of and controls from a previous study in medial orbitofrontal cortex lesion patients (Peters and D'Esposito, 2016, 2020). Finally, we focused on a simple single-parameter risky choice model (hyperbolic probability discounting; Green and Myerson, 2004), because two-parameter models (Lattimore et al., 1992; Ligneul et al., 2012) failed to converge in our data. This is likely due to the somewhat limited range of probabilities and amounts examined in our task. However, future studies would benefit from a more detailed examination of, e.g., elevation versus curvature of the probability weighting function, as dopamine has been suggested to differentially affect these processes (Burke et al., 2018; Ojala et al., 2018).

Taken together, our data extend previous investigations of modeling schemes that build on the DDM (Pedersen et al., 2017; Fontanesi et al., 2019; Peters and D'Esposito, 2020; Wagner et al., 2020), by successfully applying this approach for the first time in a clinical sample. While the data are preliminary given the small sample size, they suggest that tolcapone might impact aspects of value evidence accumulation during risky choice. However, our data do not support the idea that tolcapone attenuates risk-taking in gambling disorder. These results extend and complement previous examinations of the potential of COMT inhibition in gambling disorder (Grant et al., 2013; Kayser et al., 2017) by providing a comprehensive model-based analysis of risky decision-making.

## Synthesis

Reviewing Editor: Philippe Tobler, University of Zurich Decisions are customarily a result of the Reviewing Editor and the peer reviewers coming together and discussing their recommendations until a consensus is reached. When revisions are invited, a fact-based synthesis statement explaining their decision and outlining what is needed to prepare a revision will be listed below. The following reviewer(s) agreed to reveal their identity: Antonius Wiehler.

In this manuscript, the authors explore the effect of tolcapone (a COMT inhibitor) on risky decision-making in problem and pathological gambling (n=14). While their initial hypothesis of reduced risky decision-making under tolcapone was not confirmed, the authors found effects of tolcapone on parameters of a drift diffusion model (fitted to response times).

The topic is interesting and the rationale for exploring this particular medication in this context is largely sound. Moreover, the study is well-designed and the manuscript is well written. The investigation of tolcapone in decision-making of gambling addiction is clearly novel and worth reporting. However, the reviewers have several concerns regarding modeling, statistics and the interpretation of the results.

Main points:

1.The statistical approaches used to analyze the task are extremely convoluted and unnecessarily complicated. They cannot be checked except by someone with an extremely high level of mathematical skill and patience, well beyond probably 99.99% of the readership and researchers in general. The authors should use a simple, conventional approach - compare the main task outcome measure(s) between treatment conditions using conventional task measures and statistics. There is no need to use such complicated modelling, which is unlikely to be valid in any event, due to risk of over-fit with the small sample size, and the number of statistical assumptions made.

2.It is nice that the authors report a finding that is not supporting their hypothesis (the null effect on risky choice reduction under tolcapone). On the same line, it is interesting to see that tolcapone affects the drift parameters of the decision-making process. Nevertheless, the introduction does not lead to the use of the drift-diffusion model. Given the framing of the study, the simpler soft-max choice rule would have been sufficient. It would be of great value to the reader to learn why it could be interesting to look at the drift diffusion model in the first place.

3.Similar to the point above, the discussion about the drift diffusion model parameters seems a bit shallow. The minimum would be to provide an intuition about the behavior of the model. For example, the effect of the observed parameter changes could be explained. In the best case, there would be a discussion on why these specific changes should be linked to tolcapone and reduced frontal dopamine levels.

4.The combination of hyperbolic odds-against discounting with a non-linear DDM appears novel. However, given the effect of tolcapone on multiple parameters, the reviewers wonder whether this could be simply a compensation of parameters. For example, the drift rate max and coeff are slightly negatively correlated in the Fontanasi et al. 2019 paper. To show that parameters are indeed independent, the authors could fix one parameter at a time to the group (placebo) mean and check if the effects of tolcapone survive in the other parameters.

5.Given the initial hypothesis and the effect of tolcapone on h, it is surprising to see no model-free effect on risky choice (especially given that all participants were given identical options). One wonders to what extent this could be explained by increased choice stochasticity under tolcapone. It would be great to compare beta between conditions.

6.In the model-free analysis, Figure 1a gives the impression that RTs are shorter for risky options under tolcapone. It would be great to test the interaction of drug and choice on RT.

7.The usefulness of a comparison with control participants is clearer than the usefulness of a comparison with mOFC lesion patients (and the link to frontal dopamine levels remains somewhat obscure). The reviewers suggest to suppress the mOFC patients, to test the difference to controls more formally, and to avoid speculations about numerical differences to controls.

8.Was gambling disorder diagnosed using a clinical instrument (SOGS appears to be a screening but not a diagnostic tool)? If not please indicate as a limitation.

Smaller points:

1.Abstract. This should indicate whether findings are significant or not, not just Cohen’s D. If findings are not significant, they should not be interpreted as “increases” or “decreases” versus placebo as a reader would reasonably assume this to mean statistically significant.

2.Abstract should indicate this is single dose - at present it reads like a treatment study.

3.Risky choice task. When describing this task please provide details of previous validation studies. It would be helpful to have a figure illustrating the task to help the reader.

4."gamblers also consistently show changes in the dopamine system.” This seems over-stated. The authors seem to be referring to a small number of studies that are not consistent; these cannot be generalized to gambling in general. Alternatively, the previous literature should be reviewed more systematically

5.The paper refers to pathological and problem gamblers. However, does SOGS > 5 not indicate probable pathological gambling? It would help to clarify for readers not so familiar with the tools and terminology. Also, please use the term “gambling disorder” where appropriate because pathological gambling is not the current term used for the DSM.

6.The formatting of the citations in the text is sometimes off. For example, line 2, citation “The Lancet, 2017"; line 3, “n.d.” is not defined; line 4 - gap prior to reference starting, typo at the end”. These are just some examples.

7.The reviewers suggest moving up-front that subjects undertook MINI and what disorders were excluded.

8.Relatedly, can a subject with a BDI score of 27 be said to be free from depression? (Table 1; and results). Please clarify BDI cut-off used to help rule out depression. Or if subjects did not have depression on MINI, please clarify how they could have such high BDI score(s). Also please move the MINI up earlier so the reader knows this was done - it comes quite late in the text at present but is a positive feature of the study.

9.Please clarify whether subjects were free from alcohol use disorder. Scores on the audit seem quite high. The reviewers appreciate that the authors did breath tests but people could still have alcohol use disorder even if this test is negative (probably most would be negative if tested in the morning).

10.Peak plasma level for tolcapone is typically 1.5h. Pleas clarify why testing was done at 3h. If this was part of a study examining other measures too, please include brief information about the other procedures conducted - even if the details/results on these are to be reported separately.

11."subjects could not reliably differentiate tolcapone from placebo.” How was this determined?

12.Lines 180 and 187: Either use “action selection” or “choice rule” in the two headlines, to stay consistent

13.Line 180: there could be a quick introduction that there will be 2 different approaches used to link the subjective values to behavior: softmax for static choices, DDM for both choice and RT at the same time

14.Figure 1: It might be helpful for the reader to explain the negative RT coding again in the caption

15.Figure 3: It might be helpful to use the same x-axis for all participants to compare effects more easily

16.Figure 4 could be split into two figures, the relation to healthy controls and the tolcapone effect. Given that this is the central finding of the study please also enlarge the size of the difference plots

17.Figure 4c: the dashed line to indicate non-bias seems to be visually too similar to the indication of the group difference in the plot below. Either delete this line or make it easier to distinguish from the group difference.

18.Figures 5, 6, and 7 could be moved to the supplement

19.Table 1. Please ensure all abbreviations are spelled out (YoE doesn’t seem to be spelled out in full in the table footer)

20.Line 458: Do the parameters vary with age? This could be tested, there is no need for speculation

21.Line 463 “numerically closer to the patient’s bias” is speculation which could be tested/qualified statistically (but see the comment about the mOFC patients above).

22.Line 495: The trade-off could be tested with the procedure described above

Line 527: The gender balance seems to be a sampling bias, as the majority of patients in the population are male. That should be discussed.
